# The Eukaryotic Promoter Database: expansion of EPDnew and new promoter analysis tools

**DOI:** 10.1093/nar/gku1111

**Published:** 2014-11-06

**Authors:** René Dreos, Giovanna Ambrosini, Rouayda Cavin Périer, Philipp Bucher

**Affiliations:** 1 Swiss Institute of Bioinformatics (SIB), CH-1015 Lausanne, Switzerland; 2 Swiss Institute for Experimental Cancer Research (ISREC), School of Life Sciences, Swiss Federal Institute of Technology (EPFL), CH-1015 Lausanne, Switzerland

## Abstract

We present an update of EPDNew (http://epd.vital-it.ch), a recently introduced new part of the Eukaryotic Promoter Database (EPD) which has been described in more detail in a previous NAR Database Issue. EPD is an old database of experimentally characterized eukaryotic POL II promoters, which are conceptually defined as transcription initiation sites or regions. EPDnew is a collection of automatically compiled, organism-specific promoter lists complementing the old corpus of manually compiled promoter entries of EPD. This new part is exclusively derived from next generation sequencing data from high-throughput promoter mapping experiments. We report on the recent growth of EPDnew, its extension to additional model organisms and its improved integration with other bioinformatics resources developed by our group, in particular the Signal Search Analysis and ChIP-Seq web servers.

## INTRODUCTION

The Eukaryotic Promoter Database (EPD) is an old promoter resource first published as a table in a journal article (1). Updated versions of this promoter compilation were later distributed in machine-readable form, first on magnetic tapes and later via the Internet. A complete description of the scope, contents, format and maintenance procedures can be found in (2).

We were able to keep the basic format of EPD unchanged for almost three decades because we anticipated several future developments in its original design. For instance, we were aware of the fact that many promoters have multiple initiation sites spread over regions of variable size. We therefore distinguished from the very beginning three promoter classes called ‘single’, ‘multiple’ and ‘region’. Nevertheless, each promoter was represented by a single representative transcription start site (TSS) regardless of the class. It is further noteworthy that EPD was designed as a genome annotation database not as a sequence database. Promoters were defined by references to positions in nucleotide sequence database entries and these positions were verified and adjusted if necessary whenever the corresponding sequence entries were updated. The mechanism used for this purpose is analogous to the batch coordinate conversion method implemented in the liftOver program from the UCSC genome browser (3).

EPD was initially a manually compiled and curated database. The selection of the representative TSS was based on visual inspection of TSS mapping data published in journal articles, often in pictorial form. The TSS mapping methods used at that time were targeted at one gene at a time. In the early 2000s, novel high-throughput protocols were invented for comprehensive TSS mapping of a whole transcriptome at once (4,5). The DDBJ and EMBL nucleotide sequence libraries introduced a new data division called MGA (Mass sequences for Genome Annotation) specifically for this type of data (6). We reacted to this trend by introducing automatic procedures for inferring promoter positions from electronically disseminated public data (7) that were used in parallel with scientific literature screening. While the MGA divisions of the nucleotide sequence libraries have been superseded by the Sequence Read Archive (SRA) and European Nucleotide Archive (ENA) sequence read archives (8), we still use the term MGA in the context of EPD.

The advent of the so-called next generation sequencing technologies led to the next quantum leap in transcript mapping data production. At this time, we realized that the manual data acquisition and curation procedures upon which EPD relied for so many years were no longer sustainable. We thus revised our data acquisition strategy from scratch and created the successor database EPDnew. The first version of EPDnew was released in 2011. Since then, the old EPD database has been maintained in a frozen state. Modifications of EPD are restricted to liftOver-type operations in response to changes in nucleotide sequence entries.

EPDnew has now become a consolidated database in its productive phase. A comprehensive description of EPDnew has been presented previously (9). For users familiar with the old EPD database, we will briefly outline the main differences between the two resources (9). EPD is organized as a single file containing 4806 promoter entries from 139 different species. EPDnew is split over multiple files, each corresponding to a single model organism. In EPD, individual entries have been updated in response to new data independently of other entries. In EPDnew, an entire new version for a particular model organism is automatically generated from scratch when a new compendium of high-throughput transcript mapping data becomes available. EPD includes promoters of structural RNA genes transcribed by POL II whereas EPDNew is currently restricted to protein-coding genes present in a gene catalog from external annotation resource (Table 1). The TSS position pointers in EPD point to traditional sequence entries from Genbank, EMBL-Bank and DDBJ (10) as well as to genome sequences from RefSeq (11), whereas a promoter collection from EPDNew exclusively refers to sequences from a single genome assembly.

**Table 1.** Current contents of EPDnew

Organism, version	Assembly	Promoters	Genes	Gene catalog
*Homo sapiens* (3)	hg19	23 360	16 599 (89%)	UCSC known Genes (Mar 2009)
*Mus musculus* (2)	mm9	21 239	17 565 (90%)	UCSC known Genes (Mar 2011)
*D. melanogaster* (2)	dm3	15 073	12 603 (92%)	ENSEMBL 70
*D. rerio* (1)	danRer7	10 728	10 235 (43%)	ENSEMBL 75
*C. elegans* (1)	ce6	7120	6 363 (32%)	WormBase (WS220)

Maintaining high quality in the automatically compiled TSS collections of EPDnew is one of our prime objectives. The quality control procedures applied to this end were described in detail before (9). Very briefly, the percentage of false positives and the accuracy of TSS mapping are estimated by the enrichment and positional distribution of common promoter motifs in the corresponding promoter regions. The quality control reports resulting from such an analysis are posted on the EPD web server for each new version of EPDnew. According to these reports, promoters in EPDnew are of roughly equal quality as the manually compiled promoters of the old EPD database.

## RECENT DEVELOPMENTS

### Growth of EPDnew and extension to novel model organisms

The content of EPDnew has substantially increased over the last two years. In our previous paper (9), we presented promoter collections for three model organisms (human, mouse and *D. melanogaster*) totaling together 30 878 entries. In the meantime, the number of promoters for the two mammalian species has more than doubled, now covering about 90% of known protein-coding genes (Table 1). In addition, we were able to extend EPDnew to two new model organisms: zebrafish (*Danio rerio*) and worm (*Caenorhabditis elegans*).

The source data (12–15), from which the current versions of EPDnew were derived, are listed in Table 2. Note that the substantial growth of EPDnew is the consequence of a massive release of new TSS mapping data, which we swiftly imported into the MGA repository (9). The MGA repository is a local archive of quality-filtered and uniformly formatted functional genomics data downloaded from primary sources. An overview of its current contents is given in Table 3. The MGA repository can be viewed as the data back-end of EPD and the accessory bioinformatics web servers developed by our group. Only a small fraction of the data, primarily from the RNA-Seq class, was actually used for the automatic generation of the current EPDnew promoter collections. However, the recent addition of large numbers of other datasets (especially ChIP-Seq samples) adds value to EPD as well, as all these samples are accessible by the EPD accessory data analysis tools described in the next section.

**Table 2.** Source data

EPDnew database	Source data: type, reference or source repository	# of libraries	total tags (millions)
*H. sapiens*	CAGE from ENCODE/RIKEN, downloaded from UCSC genome browser database (12)	148	3841
*M. musculus*	CAGE from FANTOM5 (http://fantom.gsc.riken.jp/5/))	339	6236
*D. melanogaster*	CAGE from modENCODE (ftp://data.modencode.org/) TSS-seq from Machibase (13)	57	646
*D. rerio*	CAGE from Nepal *et al*. (14), downloaded from SRA (8), ID SRA055273	12	65
*C. elegans*	GRO-cap from Kruesi *et al.* (15)	8	236

**Table 3.** Current contents of the MGA repository (# of samples)

Data type	Human	Mouse	Fly^a^	Worm^b^	Fish^c^	Yeast^d^
ChIP-Seq	4738	523	220	2	9	46
RNA-seq^e^	160	339	63	19	12	
DNase FAIRE etc.	973					
DNA methylation	12	4				
Annotations^f^	20	10	3	1	1	1
Sequence-derived^g^	13	3	1		4	
Total	5916	879	287	22	26	46

^a^*D. melanogaster*.

^b^*C. elegans*.

^c^*D. rerio* (zebrafish).

^d^*Saccharomyces cerevisiae*.

^e^only TSS mapping data.

^f^includes features derived from primary data such as published ChIP-Seq peak lists.

^g^e.g. genome conservation scores, SNPs, etc.

### EPD-linked promoter analysis and selection tools

A major effort has been undertaken to integrate EPD with web-based software tools developed by us and others. As part of this effort, we completely redesign the EPD web interface. Each organism has now its own EPDnew entry portal which features navigation buttons that will directly upload the corresponding promoter collections to the data analysis tools of the ChIP-Seq (16) and signal search analysis (SSA) servers (17). The ChIP-Seq server provides programmatic access to high-throughput chromatin profiling data from the MGA repository whereas the SSA server offers DNA motif analysis.

**Figure 1.**
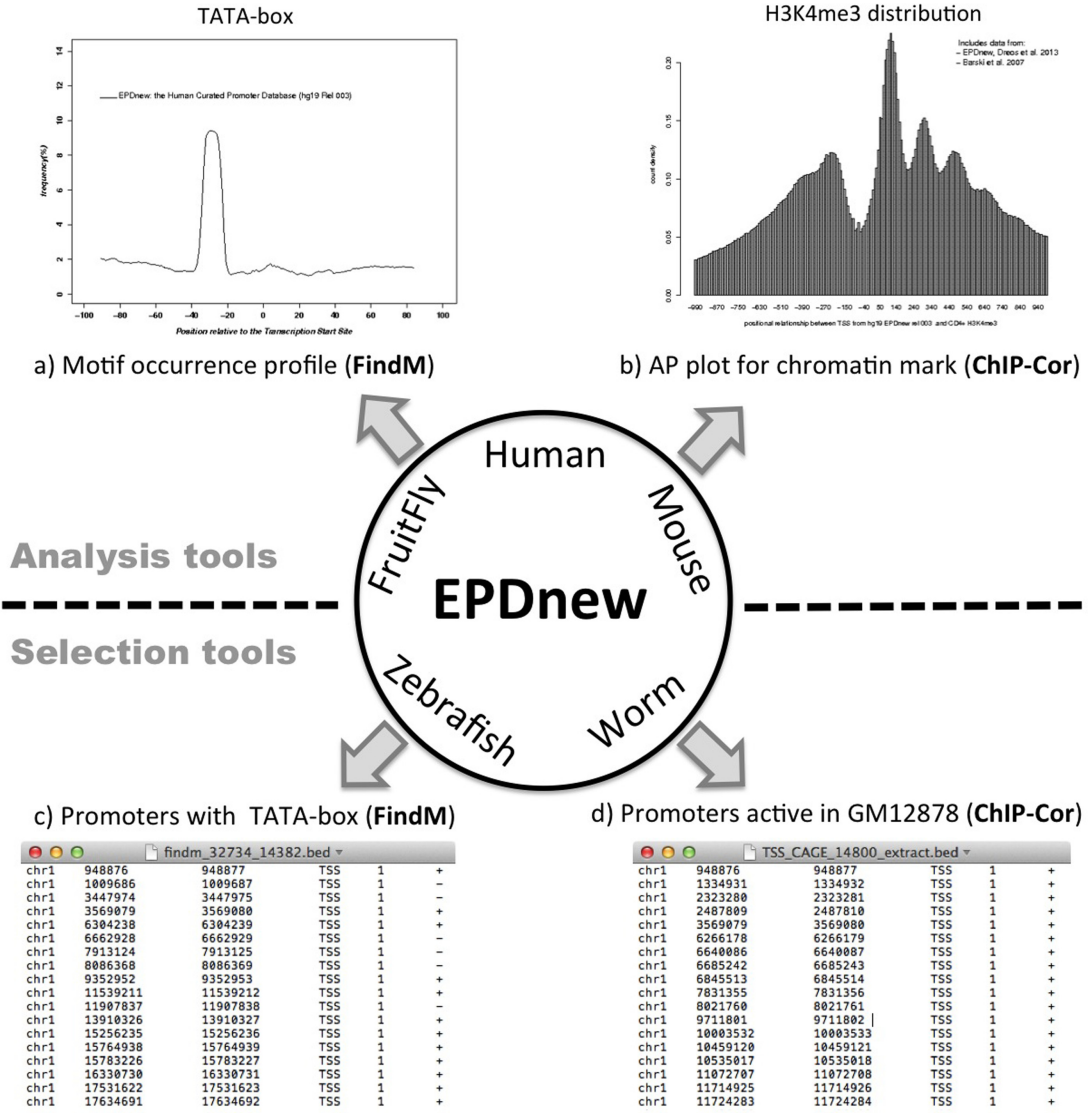
EPD analysis and selection tools. (**a**) TATA-box occurrence profile in human promoters. This picture has been obtained by following the OProf link from the human EPDnew home page and then selecting the TATA-box weight matrix from the ‘promoter motifs’ menu on the OPROF input form. (**b**) Distribution of H3K4me3-marked nucleosomes around human promoters. The figure is based on MNase-processed ChIP-Seq data from (19) stored in the MGA repository and accessible via a pull-down menu from the ChIP-Cor input form. (**c**) BED file containing genomic TSS coordinates of human promoters containing a match to the TATA-box weight matrix between positions −35 and −20 relative to the TSS. This list has been generated with the program FindM. (**d**) Genomic TSS coordinates of a promoter subset enriched in CAGE tags from lymphoblastoid cell line GM12878. This list was obtained by following the ‘ChIP-Cor’ link from the human EPDnew home page and then selecting the specific CAGE tag library as target feature via the pull-down menu on the input form. On the results page, the ‘Enriched Feature Extraction Option’ was used to select those promoters which contain at least 100 CAGE tags between positions −50 and +50 relative to the TSS given in EPDnew.

The web services directly linked to EPD perform two types of tasks: promoter analysis and subset selection. ChIP-Cor from the ChIP-Seq server is an analysis tool which generates aggregation plots (18) for two genomic features, called reference and target feature. (The generic term feature covers everything that can be mapped to a genome position, e.g. TSSs, mapped ChIP-Seq reads, etc.). The server returns a graph showing the positional distribution of the target features relative to the reference feature (see example in Figure 1b based on data from 19). The web-interface allows users to choose any sample from the MGA repository as a target or reference feature. Alternatively, features can be uploaded as a genome annotation format file in BED, GFF or BAM format. If ChIP-Cor is accessed directly from an organism-specific EPDnew home page, the corresponding promoter collection will automatically appear as the default reference feature in the ChIP-Cor input format.

The OProf (motif Occurrence Profile) tool from the SSA server performs a very similar task as ChIP-Cor. The difference is that the target feature consists of a sequence motif, which can be defined by a consensus sequence or a position-specific weight matrix (PWM). The motif occurrences are then computed on the fly by scanning the genomic sequences in the neighborhood of a reference feature defined by a so-called function positions set. The SSA server features a large collection of server-resident PWMs, selectable via a pull-down menu. Alternatively, users can paste a consensus sequence or PWM into a text area of the input form. A subset of samples from the MGA repository can be chosen as input functional position set. If OProf is accessed from an EPD page, the corresponding organism-specific promoter collection will automatically be selected as the default functional position set. An example of a motif occurrence profile generated with OProf is shown in Figure 1a.

ChIP-Cor can also be used as a subset selection tool. The results page returned by ChIP-Cor includes a small input form appearing under the heading ‘Enriched Feature Extraction Option’. This tool enables users to select those reference features (genome positions) that are covered by at least a threshold number of target feature counts within a user-defined distance range. The selected list of genomic positions is provided in several genome annotation formats, see BED file example in Figure 1d. The FindM program from the SSA server selects genomic positions on the basis of motif occurrences. It has two operational modes. In the first mode, it selects input genomic positions that are, or are not flanked by a given DNA motif within a user-defined distance range (Figure 1c). In the second mode, it searches for motifs in the neighborhood of the input positions and returns the coordinates of the found motifs. EPD further features a specialized promoter subset selection tool that allows for complex queries based on EPD annotations and a number of pre-computed features stored in a relational database.

All subset selection tools return results in several genome annotation formats. The results page further provides navigation buttons for submitting the selected subsets of genome positions to other programs of the ChIP-Seq and SSA servers, or even to external genome analysis servers, e.g. GREAT (20). In addition, the selected genomic positions can be re-mapped to another genome assembly of the same species (e.g. hg19 to hg18) or to orthologous positions in a related species (e.g. human hg19 to mouse mm9). Using these navigation buttons, complex promoter subset selection operations can be carried out by using the ChIP-Cor and/or FindM tools several times in succession.

## ACCESS

EPD and EPDnew are freely accessible without need for preregistration. Web-based access is provided via the EPD web site at http://epd.vital-it.ch/. Data files can be downloaded via FTP from ftp://ccg.vital-it.ch/.
